# Clinical study of the oral manifestations and related factors in type 2 diabetics patients

**DOI:** 10.1590/S1808-86942011000200002

**Published:** 2015-10-19

**Authors:** Maria Goretti de Menezes Sousa, Antonio de Lisboa Lopes Costa, Angelo Giuseppe Roncalli

**Affiliations:** 1 Master's degree student. State coordinator of oral health, Public Health Secretariat, RN state; 2 Doctoral degree in oral pathology. Associate professor II, UFRN; 3 Doctoral degree in social dentistry. Associate professor I, UFRN

**Keywords:** diabetes mellitus, epidemiology, mouth mucosa

## Abstract

Diabetes Mellitus (DM) is reported with and associated to oral alterations, with conflicting results. The aim of this study was to identify the prevalence of oral soft tissue alterations in type 2 diabetes mellitus patients.

**Material and Methods:**

Socioeconomic variables, gender, heredity, capillary glucose control and local factors (prosthesis, dry mouth sensation) were analyzed in 196 diabetic and non-diabetic patients enrolled in HIPERDIA, at 41 Health Units of Natal, Brazil.

**Study Design:**

A case study.

**Results:**

The last blood glucose mean was 177.0 mg/dl for diabetics and 89.46 mg/dl for non-diabetics. Mean capillary blood glucose was elevated in diabetics (215.95 mg/dl); it was 102.31 mg/dl in non-diabetics. The family history confirmed the heredity nature of the disease in 68.8% of diabetic patients (n = 66) (*p* < 0.001); salivary flow was 49% (n = 47) in diabetics, and 34% (n = 34) in non-diabetics. Candidiasis was present in 30.5% of diabetic patients (n=29) and 36% of non-diabetics (n=36). Both groups had lesions in the palate - 81.4% (n = 35) in diabetics, and 71.1% in non-diabetics (n = 27) (*p* = 0.68).

**Conclusion:**

The alterations are not related to diabetes and are present independently of having or not type 2 Diabetes Mellitus.

## INTRODUCTION

Diabetes mellitus (diabetes) is a complex metabolic disease characterized by altered carbohydrate, lipid, and protein metabolism, which results in marked or absolute insulin deficiency - type 1 diabetes - or peripheral tissue insulin resistance - type 2 diabetes. Type 3 is gestational diabetes, where there is carbohydrate intolerance during pregnancy.^1^

A Brazilian multicentre study on the prevalence of diabetes, coordinated by the Ministry o Health,^2^ has mapped the disease in this country: the prevalence is 7.6 % in the urban adult population of nine capitals. The study revealed that 46.5 % of diabetics ignored their condition, and 22.3 % had but did not treat the disease.

Faced with an increasing diabetic and hypertensive population, the Ministry of Health, in a partnership with State and Municipal Secretariats, scientific societies (diabetes, cardiology, and nephrology), and associations of diabetic and hypertensive patients, has reorganized healthcare through the Plan for Reorganizing Healthcare for Arterial Hypertension and Diabetes, to reduce the morbidity and mortality of these conditions. The plan improves healthcare for patients with these diseases by health-promoting actions involving preventive, curative and control measures.^3^

Several systemic diseases manifest in the mouth, including diabetes. Absence of metabolic control appears to alter the susceptibility of patients with diabetes to periodontal disease, fungal infections, and changes in taste. The relationship between diabetes and oral lichen planus and dental caries is less obvious; several studies have shown widely diverging results.^4^, ^5^, ^6^, ^7^

A few studies have suggested that decrease salivary flow results from the use of certain drugs, which would result in changes within the mouth, leading to caries, periodontal disease, and soft tissue alterations; the latter may foster invasion by opportunistic microorganisms. Several types of drugs may cause a subjective feeling of dry mouth, or may induce low salivary flow; these include anticholinergics, antidepressants, diuretics, antihistamines, myorelaxants, diazepinic drugs, and sympatheticomimetics such as hypotensive drugs.^8^, ^9^ This last category is commonly used by diabetics who also have arterial hypertension as a comorbidity.

On the other hand, there are published reports of salivary gland disorders as a systemic consequence of diabetes affecting the parenchyma of salivary glands, resulting in altered salivary gland function. Histological alterations in these glands change the shape and function of acinar cells, resulting in decreased enzyme activity because of degenerative complications of diabetic angiopathy, neuropathy, and hormone changes following metabolic derangement.^10^, ^11^ Murrah^12^ described the oral signs of diabetes as follows: xerostomy, angular cheilitis, decreased salivary flow, increased glucose levels in saliva produced by the parotid gland as a results of elevated blood glucose.

The scientific community has not reached any conclusion about the relation between use of dentures and oral alterations in diabetic patients. Several conflicting studies have been published on dentures as risk factors for stomatitis and candidiasis in diabetic^13^, ^14^, ^15^ and non-diabetic^16^, ^17^ patients.

The purpose of this study was to check which oral soft tissue manifestations were found in type 2 diabetes, and the correlation between these findings and this complex disease.

## MATERIALS AND METHODS

A observational individualized cross-sectional study was made from December 2007 to December 2008. The sample comprised 196 diabetic and non-diabetic patients. The sample size was calculated based on a 35 % prevalence of outcomes (oral alterations), a 20 % margin of error, and a 20 % non-response rate. The confidence level was 95 % (a=0.05).^18^

From an initial calculated sample of 220 patients, 10 % (20 persons) did not present, and 1.8 % (4 persons) decided voluntarily not to participate. Thus, the final sample consisted of 196 patients, of which 96 were diabetic and 100 were non-diabetic.

The inclusion criteria were patients of both sexes aged 40 years or over, diagnosed with type 2 diabetes, and non-diabetics of both sexes and the same age group. The exclusion criteria were patients with type 1 diabetes and subjects aged less than 40 years.

A questionnaire was applied to gather data on the clinical history, the social and economic profile,^19^ and the dental history. A glucometer (Accucheck Roche) was used to measure glucose levels (capillary glucose), which was dichotomized as follows: postprandial values ≤ 140mg/ dL - controlled glucose levels; and postprandial values ≥ 140mg/dL - uncontrolled glucose levels.^20^, ^21^ Arterial blood pressure was also measured. Two observers carried out the physical examination, which consisted of noting the status of the lips, the jugal mucosa, the tongue, the floor of the mouth, the hard and soft palates, and use of dentures. A World Health Organization (WHO) form for epidemiological studies was used.^22^ Diagnosis of different types of candidiasis was based on the clinical signs (Neville et al.).^23^ A similar procedure was adopted for the medical diagnosis of oral lichen planus, noting the presence of Wickham striae to characterize reticular lichen planus,^23^ the clinical type encountered in the sample, as well as aphthous ulcers characterized by lesions covered with white-yellowish membranes surrounded by an erythematous halo.^23^ We excluded non-pathologic or developmental alterations such as Fordyce granules, lingual varices, benign migratory glossitis, and fissured tongue.^23^

Our theoretical model used the health status of the oral mucosa as a dependent variable. Unaltered soft tissues in the mouth were considered normal, and altered soft tissues because of candidiasis, lichen planus, and aphtha were considered as an abnormal status.

Data analysis consisted of descriptive statistics of quantitative variables, the description of categorical variables based on the chi square test, and description of variables in the group of diabetic patients relative to the treatment. The statistical significance level was 5 %.

All participants were asked to sign a free informed consent form after being given detailed information about the goals of this study. The institutional review board approved the study (no. 044/05).

## RESULTS

Descriptive statistics of quantitative variables showed that the age ranged from 40 to 81 years, the mean age of non-diabetic patients was 58.2 years, and of diabetic patients, 58.9 years. The mean time elapsed until diagnosis of diabetic patients was 9.1 years. The mean blood glucose level in non-diabetic patients was 89.4 mg/ dL, and the mean blood glucose level in diabetic patients was 177 mg/dL. The mean capillary blood glucose level in non-diabetic patients was 102.3 mg/dL, and among diabetics, 215.9 mg/dL. The mean arterial systolic pressure in non-diabetics was 126.9 mmHg, and among diabetics, 132 mmHg. The mean arterial diastolic pressure in non-diabetic patients was 81 mmHg, and in diabetic patients, 83.23 mmHg. (Table 1).

**Table 1:** Descriptive statistics of quantitative variables in the study sample (2009).

Variable	Group	n	Mean	S.D.	Min	Q25	Med	Q75	Max
Age	Non-diabetics	100	58.29	10.19	31	51	60.50	65	80
	Diabetics	96	58.94	10.29	36	52	60.00	66	81
	Total	196	58.61	10.22	31	51	60.00	65	81
Time before diagnosis	Non-diabetics	-	-	-	-	-	-	-	-
	Diabetics	96	9.16	8.28	0	3	7.00	14	47
	Total	96	9.16	8.28	0	3	7.00	14	47
Last blood glucose	Non-diabetics	13	89.46	17.09	55.00	79.50	92.00	98.00	120
	Diabetics	93	177.06	82.52	70.00	117.00	146.00	219.50	443
	Total	106	166.32	82.66	55.00	106.75	137.00	209.25	443
Capillary glucose	Non-diabetics	100	102.31	19.69	70.00	90.00	100.00	112.00	215
	Diabetics	96	215.95	103.44	67.00	129.00	193.00	292.25	497
	Total	196	157.97	93.02	67.00	96.00	115.00	193.50	497
Systolic A.P.	Non-diabetics	100	126.90	18.52	80	120	120.00	140	180
	Diabetics	96	132.08	20.62	90	120	130.00	140	200
	Total	196	129.44	19.69	80	120	130.00	140	200
Diastolic A.P.	Non-diabetics	100	81.00	12.59	50	70	80.00	90	120
	Diabetics	96	83.23	14.20	50	80	80.00	90	140
	Total	196	82.09	13.41	50	73	80.00	90	140

The non-diabetic patients comprised 100 subjects, of which 27 were male (27 %) and 73 were female (73 %); among the 96 diabetic patients, 31 were male (32.3 %) and 65 were female (67.7 %). In the non-diabetic study sample, 76 were black or brown (76.0 %) and 24 were white (24.0 %); in the sample of diabetics, 70 were black or brown (72.9 %) and 26 were white (72.9 %). The social and economic status of non-diabetics included 28 subjects (28.0 %) with higher income levels (classes A2, B1, B2, and C), and 72 (72.0 %) subjects with lower income levels (D and E). In diabetic patients, 26 subjects had higher income levels (27.1 %) and 70 subjects had lower income levels (72.9 %).

The family history of non-diabetics showed that there were 42 patients (42.0 %) with a history of diabetes in family members and 58 without any history of family members diagnosed with diabetes (58.0 %). The family history of diabetics showed that there were 66 patients (68.8 %) with a history of diabetes in family members and 30 without any history of family members diagnosed with diabetes (31.2 %), which was statistically significant (*p*<0.001). There were 29 non-diabetic patients (63.0 %) in the relationship with parents category (degree of relationship with parents) and 17 (37.0 %) in the relationship with siblings category. There were 40 diabetic patients (59.7 %) in the relationship with parents category and 27 (40.3 %) in the relationship with siblings category.

The results of variables attributed to risk factors for the presence of arterial hypertension was that among non-diabetics there were 62 hypertensive patients (62.0 %) and 38 non-hypertensive patients (38.0 %). Among diabetics, there were 59 hypertensive patients (61.5 %) and 37 non-hypertensive patients (38.5 %).

The habit of smoking did not affect 76 non-diabetic patients (76.0 %) who had not smoked for at least ten years, and therefore was considered a favorable effect, compared to 24 non-diabetic patients (24.0 %) who smoked or had ceased smoking less than ten years ago, in whom it was considered an unfavorable effect. Among diabetic patients, these numbers were 74 (77.1 %) - favorable - and 22 (22.9 %) - unfavorable.

Decreased salivary flow was present in 34 non-diabetic patients (34.0 %); salivary flow was normal in 66 non-diabetic patients (66.0 %). Decreased salivary flow was present in 47 diabetic patients (49.0 %); salivary flow was normal in 49 diabetic patients (51.0 %).

Oral health (soft tissue examination) was normal in 58 non-diabetic patients (58.0 %); 36 non-diabetic patients (36.0 %) had candidiasis (palatal prosthetic stomatitis and commissural angular cheilitis), and 6 non-diabetic patients (6.0 %) had other conditions (lichen planus and aphtha). Oral health was normal in 61 diabetic patients (64.2 %); 29 diabetic patients (30.5 %) had candidiasis (palatal prosthetic stomatitis and commissural angular cheilitis), and 6 diabetic patients (5.3 %) had other conditions (lichen planus and aphtha).

The site of oral conditions in non-diabetic patients was the hard palate in 35 patients (81.4 %), the jugal mucosa in 4 patients (9.4 %), and other sites in another 4 patients (9.4 %). The site of oral conditions in diabetic patients was the hard palate in 27 patients (71.1 %), the jugal mucosa in 2 patients (5.8 %), and other sites in 8 patients (23.1 %).

Full upper dentures were used by 52 non-diabetic patients (52.0 %); 48 non-diabetic patients (48.0 %) did not use full dentures or used other types of dental prosthetic appliances. Full upper dentures were used by 50 diabetic patients (52.1 %); 46 diabetic patients did not use full upper dentures or used other types of dental prosthetic appliances (47.9 %).

In 52 non-diabetic patients that used dentures, 36 had candidiasis (36 %). In 50 diabetic patients that used dentures (52.1 %), 29 had candidiasis (30.5).

There were 20 non-diabetic patients (20.0 %) that used complete lower dentures; 80 non-diabetics did not use dental prosthetic appliances (80.0 %). There were 18 diabetic patients (18.8 %) with full lower dentures; 78 diabetics did not use dental prosthetic appliances (81.2 %) (Table 2).

**Table 2:** Description of categorical variables in the study sample (2009).

		Non-diabetics		Diabetics		Total		p*
		n	%	n	%	n	%	
Social and demographic								
	Male	27	27.0	31	32.3	58	29.6	
Sex	Female	73	73.0	65	67.7	138	70.4	0,41
	Total	100	100.0	96	100.0	196	100.0	
	Black and Brown	76	76.0	70	72.9	146	74.5	
Skin color	White	24	24.0	26	27.1	50	25.5	0,62
	Total	100	100.0	96	100.0	196	100.0	
Social class	Classes A2. B1. B2. and C	28	28.0	26	27.1	54	27.6	
	Classes D and E	72	72.0	70	72.9	142	72.4	0,88
	Total	100	100.0	96	100.0	196	100.0	

Diabetes								

Family history	Yes	42	42.0	66	68.8	108	55.1	
	No	58	58.0	30	31.2	88	44.9	*p*<0,001
	Total	100	100.0	96	100.0	196	100.0	
Relationship	Parents	29	63.0	40	59.7	69	61.1	
	Siblings	17	37.0	27	40.3	44	38.9	0,72
	Total	46	100.0	67	100.0	113	100.0	

Risk factors								

Arterial hypertension	Yes	62	62.0	59	61.5	121	61.7	
	No	38	38.0	37	38.5	75	38.3	0,93
	Total	100	100.0	96	100.0	196	100.0	
Smoking	Favorable	76	76.0	74	77.1	150	76.5	
	Unfavorable	24	24.0	22	22.9	46	23.5	0,85
	Total	100	100.0	96	100.0	196	100.0	
Decreased salivary flow	Yes	34	34.0	47	49.0	81	41.3	
	No	66	66.0	49	51.0	115	58.7	0,03
	Total	100	100.0	96	100.0	196	100.0	

Status of oral health								

Exam of soft tissues	Normal	58	58.0	61	64.2	119	61.0	
	Candidiasis	36	36.0	29	30.5	65	33.3	0,23
	Others **	6	6.0	6	5.3	11	5.7	
	Total	100	100.0	96	100.0	196	100.0	
Site	Palate	35	81.4	27	71.1	62	76.5	
	Jugal mucosa	4	9.3	2	5.8	6	7.5	0,68
	Others ***	4	9.3	8	23.1	13	16.0	
	Total	43	100.0	38	100.0	81	100.0	
Use of upper denture	Denture Total	52	52.0	50	52.1	102	52.0	
	Other/Not uses	48	48.0	46	47.9	94	48.0	0,39
	Total	100	100.0	96	100.0	196	100.0	
Use of lower denture	Denture Total	20	20.0	18	18.8	38	19.4	
	Other/Not uses	80	80.0	78	81.2	158	80.6	0,14
	Total	100	100.0	96	100.0	196	100.0	

* Based on the chi square test

** Lichen planus and aphtha

*** Labial commissure, tongue, and alveolar/gingival margins

Analysis of diabetics and type of treatment showed that in diabetic patients aged 60 years or less 37 diabetic patients (72.5 %) were monitored monthly, and 14 diabetic patients (27.5 %) were not monitored monthly. There were 30 diabetics (66.7 %) aged over 60 years that were monitored monthly; 15 diabetics (33.3 %) were not monitored monthly.

In the group aged 60 years or less, only 12 patients (23.5 %) used insulin; 39 patients (76.5 %) were not using insulin. In the group aged over 60 years, 8 patients (17.8 %) were using insulin, and 37 (82.2 %) were not using insulin.

There were 40 patients (78.4 %) taking oral hypoglycemic drugs in the group aged 60 years or less; 11 patients in this group (21.6 %) were not taking hypoglycemic medication. In the group aged over 60 years, 39 (86.7 %) were taking oral hypoglycemic drugs, and only 6 patients (13.3 %) were not taking hypoglycemic medication.

In the group aged 60 years or less, 34 patients (66.7 %) were on diets, while 17 patients (33.3 %) did not follow any diet. In the group aged over 60 years, 38 patients (84.4 %) were on diets, while 7 patients (15.6 %) did not follow any diet (Table 3).

**Table 3:** Description of treatment variables in the group of diabetics (2009)

		≤ 60 years		> 60 years		Total		p^*^
		n	%	n	%	n	%	
Monthly monitoring	Yes	37	72.5	30	66.7	67	69.8	
	No	14	27.5	15	33.3	29	30.2	0.53
	Total	51	100.0	45	100.0	96	100.0	
Use of insulin	Yes	12	23.5	8	17.8	20	20.8	
	No	39	76.5	37	82.2	76	79.2	0.48
	Total	51	100.0	45	100.0	96	100.0	
Use of hypoglycemic drugs	Yes	40	78.4	39	86.7	79	82.3	
	No	11	21.6	6	13.3	17	17.7	0.29
	Total	51	100.0	45	100.0	96	100.0	
Use of diet	Yes	34	66.7	38	84.4	72	75.0	
	No	17	33.3	7	15.6	24	25.0	0.45
	Total	51	100.0	45	100.0	96	100.0	

## DISCUSSION

Several systemic conditions cause well defined effects in the mouth; many of these alterations are pathognomonic of the main disease, and have been well studied. In other diseases, such as diabetes, the relation with oral manifestations of the disease remains controversial.

The two study sample groups (diabetics and non-diabetics) did not differ statistically relative to age, sex, skin color, and income level, which characterized type 2 diabetes as being independent of the social and demographic status of the general population; the disease is present in all social classes.^2^, ^24^, ^25^

The mean capillary glucose level was high (215.9 mg/dL), even with many patients using oral hypoglycemic drugs and insulin. It is interesting to note that irrespective of positive answers for monthly monitoring at basic healthcare units and following diets, patients were unable to attain adequate control of blood glucose levels; thus, more effective action is required. Furthermore, there are known difficulties in managing blood glucose levels in diabetics because of the complex nature of this disease.

The aim of management is to effectively control the disease in such individuals;^26^ if uncontrolled, diabetes causes systemic complications because of long-term hyperglycemia. Our results relative to blood glucose levels in diabetic patients concur with those of Guggenheimer,^15^ Manfredi^7^ and Carvalho.^27^ According to the American Diabetes Association,^28^ controlling blood glucose is essential for managing diabetes; such control is associated with a reduced rate of several systemic complications.

In our study sample, diabetic patients and non-diabetics could present arterial hypertension or not. The results showing control of arterial systolic and diastolic pressure to normal limits were distributed homogeneously in both groups. Diabetes is an independent risk factor for microvascular disease; it is commonly associated with elevated arterial pressure. Thus, controlling blood glucose helps avoid complications of diabetes in the mouth,^10^, ^13^, ^14^, ^24^ cardiovascular diseases, retinopathy, and nephropathy.^28^

The literature shows that the family history of diabetics has been widely studied to find inheritance patterns in this disease, around which researchers agree. We found a positive association for a family history of diabetes: 66 patients (68.8 %) had diabetic family members, mostly parents (father or mother). These findings are similar to those of Goldenberg,^25^ Crispim^29^ and Goldenberg.^30^

Smoking in our sample included a ’favorable’ category (patients who had stopped smoking for more than 10 years). This variable was positive in interviewees that confirmed cessation of smoking in this sample of adult and elderly subjects. Smoking has been related to vascular complications in diabetic and hypertensive patients; encouraging cessation of smoking is one of the measures for these groups, although the consequences of smoking are similar to those in the general population.^29^

Studies on salivary flow have shown conflicting results because of the different methods that have been used - measurements of saliva at rest, stimulated salivary flow, self-reported decreased salivary flow in diabetics and non-diabetics. We found more diabetic patients with decreased salivary flow compared to non-diabetic patients. Altered salivary flow in diabetic patients has been attributed to changes in the parenchyma of salivary glands,^12^ and to complications of the disease - neuropathy and angiopathy.^10^, ^11^ Quirino et al.^14^ reported different results in an analysis of diabetic patients with controlled and uncontrolled blood glucose levels; they reported that decreased salivary flow was present in the uncontrolled group. Chávez et al.^10^ found no statistical significance in salivary flow patterns between well-controlled diabetic patients and a control group of non-diabetics.

Features of healthy soft tissues were present in diabetics and non-diabetics. We found no association between candidiasis and type 2 diabetes in our sample; it was equally present in both groups. We suggest that there is a need for a better definition of the host-parasite relationship. Oral candidiasis has been related to several factors, including diabetes. These factors, as mentioned in the literature, include decreased salivary flow in diabetic patients because of altered salivary glands,^12^ altered glucose levels in saliva that could facilitate adhesion of *C. albicans* to oral tissues,^31^, ^30^ uncontrolled blood glucose levels,^30^ and use of dentures or poorly fitting dental appliances;^15^, ^16^, ^32^, ^24^ these factors would not act in isolation, but rather as a set of risk factors.^14^, ^31^, ^33^

The palate is the most frequent site of these changes in both groups; it appears to be related more strongly with the presence of full upper dentures^13^, ^24^ as a possible predisposing factor for candidiasis. This infection was present in diabetic and non-diabetic subjects. The area under the dentures would be more susceptible to fungal infection because of poor host defense due to lack of salivary factors.^16^ The microorganisms would form a biofilm that facilitates adhesion, as a first step for fungal infection.^32^ Angular cheilitis was directly related with the type and time of use of dentures - lack of adequate vertical dimension and quality of dentures.

Our results are similar to those of Quirino et al.^14^ and Shulman et al.^16^ The latter, in a study of risk factors for denture-associated stomatitis and candidiasis of 3,450 adults, found no association between diabetes and changes in soft tissues of the mouth because of full dentures. De Lima et al.^17^ compared the denture-associated oral manifestations in diabetic and non-diabetic patients and found more oral lesions in non-diabetic patients.

Concerning the treatment variables, monthly followup at healthcare units consists of delivering medication and assessing the health status in general, including measuring arterial blood pressure, checking blood glucose levels, and identifying patients not responding to the prescribed regimen; these patients are monitored for more effective control.^26^

We found that adhesion to outpatient care at basic healthcare units - in which the Family Health Program is conducted - was significant in both groups. Monthly visits include discussing healthcare measures for diabetes and high blood pressure, although blood glucose levels are not monitored at these units, which lack glucometers. Return visits depend on physicians.

The variables on insulin and hypoglycemic medication showed that the treatment of choice for diabetics was oral hypoglycemic drugs, to which diet was added for controlling the disease in both groups. Nevertheless, we found that capillary glucose levels were elevated in diabetic patients; this suggested the need for reassessing the prescribed treatment and stimulating more rigorous diets and exercise.^28^, ^34^ Appropriate medication - sulphonylureas, biguanide, or insulin - as needed for controlling glucose levels in these patients.^34^

## CONCLUSION

The findings of this study were unrelated to the presence or absence of type 2 diabetes; there are several factors that may give rise to these changes in the oral cavity, one of them being the use of dental prosthetic appliances.
